# Comparison of the gut microbiota of people in France and Saudi Arabia

**DOI:** 10.1038/nutd.2015.3

**Published:** 2015-04-27

**Authors:** M Yasir, E Angelakis, F Bibi, E I Azhar, D Bachar, J-C Lagier, B Gaborit, A M Hassan, A A Jiman-Fatani, K Z Alshali, C Robert, A Dutour, D Raoult

**Affiliations:** 1 Special Infectious Agents Unit, King Fahd Medical Research Center, King Abdulaziz University, Jeddah, Saudi Arabia; 2 Unité de Recherche sur les Maladies Infectieuses et Tropicales Emergentes: URMITE CNRS-IRD 198 UMR 6236, Faculté de Médecine, Université de la Méditerranée, Marseille, France; 3 Department of Medical Laboratory Technology, Faculty of Applied Medical Sciences, King Abdulaziz University, Jeddah, Saudi Arabia; 4 Department of Endocrinology, Metabolic Diseases and Nutrition, CHU Nord, Marseille, France; 5 Department of Medical Microbiology and Parasitology, Faculty of Medicine, King Abdulaziz University, Jeddah, Saudi Arabia; 6 Department of Medicine, Faculty of Medicine, King Abdulaziz University, Jeddah, Saudi Arabia

## Abstract

**Background/Objectives:**

The gut microbiota contributes to energy acquisition from food, and changes in the gut microbiome are associated with obesity. The eating habits of Saudis are much different than those of Europeans, and our objective was to compare the fecal microbiota of obese and normal weight Saudis and French.

**Subjects/Methods:**

Illumina MiSeq deep sequencing was used to test the gut microbiota of 9 normal weight and 9 obese individuals from Saudi Arabia and 16 normal weight and 12 obese individuals from France.

**Results:**

Obese French possessed significantly more relative *Proteobacteria* (*P*=0.002) and *Bacteroidetes* (*P*=0.05) and had lower richness and biodiversity at all the operational taxonomic unit (OTU) cutoffs (*P*<0.05) than normal weight French. Obese Saudis possessed significantly more *Firmicutes* (*P*=0.001) without a difference in richness (*P*=0.2) and biodiversity (*P*=0.3) compared with normal weight Saudis. We found a common bacterial species core of 23 species existing in ⩾50% of obese and normal weight Saudis and 29 species in ⩾50% of obese and normal weight French. *Actinomyces odontolyticus*, *Escherichia coli* and *Ruminococcus obeum* were present in at least 50% of all individuals tested. French individuals had significantly higher richness and biodiversity compared with Saudis at all the OTU cutoffs (*P*<0.05).

**Conclusion:**

Microbiota differences between obese and normal weight French were not similar to those between obese and normal weight Saudis. The studies of different populations can result in contrasting data regarding the associations of the gut microbiota and obesity.

## Introduction

Obesity is a chronic disease that is defined as an excessive accumulation of fat mass in the body due to both environmental and genetic factors.^1^ Obesity is a risk factor of several diseases, such as type 2 diabetes, hypertension and cancer.^2, 3^ The World Health Organization has estimated that >1.4 billion adults (above the age of 15 years) were overweight. Among those, 400 million were obese, and approximately 2.8 million people die every year owing to obesity-related disorders. There is evidence that the gut microbiota contributes to energy acquisition from food, and changes in the gut microbiome may be associated with obesity.^1, 4, 5^ Moreover, recent lifestyle innovations, most notably the ‘Western' diet, have altered the metabolic activity of our resident gut microbiota, and these changes are suspected of contributing to obesity.^4, 5, 6^ Gut microbiota modifications by antibiotics and probiotics have also been associated with weight modifications.^5, 7^

To understand the impact of the gut microbiota on human health and well-being, it is necessary to decipher the content, diversity and function of the microbial gut community.^8^ We do not yet completely understand how the different environments and diets around the world have affected the microbial ecology of the human gut microbiota. Recent advancements in high-throughput technologies have greatly revolutionized our knowledge of the gut microbiota and revealed a substantial diversity of the gut microbiota between individuals from different countries.^9, 10, 11^ The existence of three enterotypes in the human gut microbiome that vary in species and functional composition was recently demonstrated using data that spans several nations and continents.^11^ The kingdom of Saudi Arabia is considered one of the most rapidly growing economies of the world where obesity is rapidly increasing and becoming an alarming public health concern. It is estimated that 14% of adult males and 24% of females are obese in Saudi Arabia.^12, 13^ Eating habits of Saudis are much different than those of European countries, with a very limited variety of foods and an absence of fruits and vegetables.^12^ In France, the prevalence of obesity among adults was relatively stable between 1980 and 1991, but recent surveys have highlighted a sharp increase over the period 1997–2006.^14^ In addition, obesity has been associated with a decrease in the *Firmicutes/Bacteroidetes* ratio,^15^ and obese individuals have been shown to harbor a less diverse bacterial population than lean individuals.^8, 15^ Sampling a population of humans representing different cultural traditions offers an opportunity to discover how our gut microbiomes vary between populations and respond to our changing lifestyles. As a result, our aim was to use the Illumina MiSeq deep sequencing platform (Illumina, San Diego, CA, USA) to determine the gut microbiome of the Saudi population in the context of obesity and to compare it with that of French participants.

## Materials and methods

### Subject selection criteria

This study protocol was approved by the Ethics Committee of the King AbdulAziz University under agreement number (014-CEGMR-2-ETH-P) and by the Ethics Committee of the Institut Fédératif de Recherche IFR48, Faculty of Medicine, Marseille, France. The agreement of the ethics committee of the IFR48 (Marseille, France) was obtained under reference 09–022. Informed consent forms were provided to all participants and obtained at the time of sample collection. In Saudi Arabia, we tested normal weight and obese male volunteers living in urban areas. In France, we tested normal weight and obese individuals from urban areas. The exclusion criteria were individuals aged <18 years, history of colon cancer, inflammatory bowel disease, acute or chronic diarrhea in the previous 8 weeks and treatment with an antibiotic in the 6 months before fecal sampling. Stool samples were collected under aseptic conditions with clean, dry screw-top containers and immediately stored at −20 °C. Normal weight was defined as individuals with a body mass index (BMI) of 20–25 kg m^−^^2^, and obese was defined as people with a BMI>30 kg m^−^^2^.

### Extraction of DNA from stool samples and 16S rRNA sequencing using MiSeq technology

Fecal DNA was extracted from samples using the NucleoSpin Tissue Mini Kit (Macherey Nagel, Hoerdt, France) according to a previously described protocol.^16^ Samples were sequenced for 16S rRNA sequencing using MiSeq technology. PCR amplified templates out of genomic DNA using the surrounding conserved regions' V3–V4 primers with overhang adapters (FwOvAd_341F TCGTCGGCAGCGTCAGATGTGTATAAGAGACAGCCTACGGGNGGCWGCAG; ReOvAd_785RGTCTCGTGGGCTCGGAGATGTGTATAAGAGACAGGACTACHVGGGTATCTAATCC). Samples were amplified individually for the 16S ‘V3–V4' regions by the taq Phusion (Thermo Fisher Scientific, Waltham, MA, USA) and visualized on the Caliper LabchipII device (Illumina) by a DNA 1K Labchip. After purification on Ampure beads (Thermo Fisher Scientific), the concentrations were measured using high-sensitivity Qubit technology (Thermo Fisher Scientific). Using a subsequent limited cycle PCR on 1 ng of each PCR product, Illumina sequencing adapters and dual-index barcodes were added to each amplicon. After purification on Ampure beads, the libraries were then normalized according to the Nextera XT (Illumina) protocol. The 96 multiplexed samples were pooled into a single library for sequencing on the MiSeq. The pooled library containing indexed amplicons was loaded onto the reagent cartridge and then onto the instrument along with the flow cell. Automated cluster generation and paired-end sequencing with dual index reads was performed in a single 39-h run in a 2 × 250 bp. On the instrument, the global cluster density and the global passed filter per flowcell were generated. The MiSeq Reporter software (Illumina) determined the percentage of indexing and cluster passed filter for each amplicon or library. The raw data were configured in fastaq files for R1 and R2 reads.

### Data processing: filtering the reads, dereplication and clustering

From the raw fastq files supplied by Illumina Miseq, the Paired End sequences were assembled using pandaseq.^17^ Sequences were then extracted from the fasta file (produced by pandaseq) only if the sequences contained the primers that were used in PCR amplification. In the next step, all the sequences containing *N* and the sequences shorter than 200 nts were removed. The primers were also removed from the sequences. After these filtering steps, the high-quality sequences were strictly dereplicated (clustering of duplicate sequences) and were sorted by decreasing number of abundance.^18^ These sequences in sorted order were then clustered at *k*=10 (⩾97% identity) number of differences as described previously.^18, 19^ Next, the extraction of operational taxonomic units (OTUs; representative sequences of each cluster) was performed, where the representative OTUs are the unique sequences from each cluster that has the maximum number of occurrences during the PCR amplification.^18, 19^ The abundance information of each sequence was calculated during the strict dereplication step as described earlier.

### Building the reference database

We built our local reference database. First, the release 115 of the SILVA SSU and LSU database^20^ was downloaded and from this a local database of predicted amplicon sequences was built. During the construction of our local reference database, we considered only those SILVA SSU reference sequences containing the two primers (which were used in the PCR amplifications), by allowing three differences between each primer and the SILVA reference sequences.^20^ Finally, our local reference database contained a total of 479 927 sequences.

### Taxonomic assignments

The OTUs (representative sequence from each OTU) extracted in the previous step were searched against our local reference database by using a Needleman–Wunsch global alignment algorithm. The best matches >80% similarity with each of the unique sequences were extracted from the reference database. Sorting of these extracted reference sequences was then performed according to the decreasing percentage of similarity, and the fractional values were rounded to an integer. The reference sequences with the highest percentage of similarity with the OTUs were used for taxonomic assignments, and taxonomy to each rank was obtained by the consensus of these taxonomies when there were more results with same percentage of similarity. For example, a tag with 98% similarity to the class *Gammaproteobacteria* and *Alphaproteobacteria* was only assigned to the phylum *Proteobacteria*. When similarity was 80%, sequences were not assigned.^18, 20^ Finally, all the tags were clustered to different taxon ranks according to the consensus taxonomy of the unique tags (representative of each OTUs). Principal Coordinates Analysis was calculated in QIIME by choosing Bray–Curtis distance methods at the genus level.

### Statistical analysis

We calculated the richness and biodiversity index of the OTUs by using the mothur software package^21^ with the implementation of the Chao1 and non-parametric Shannon formula.^22^ We estimated richness using the Chao1 index and diversity, which depends on how uniformly sequences are spread in different OTUs using the non-parametric Shannon formula. One-way analysis of variance and Tukey's HSD (Honestly Significant Difference) tests were used to statistically analyze BMI, age of the participants, OUTs and the Chao1 and Shannon index from sequence reads of all individuals. Non-parametric Kruskal–Wallis along with Mann–Whitney analyses were performed to identify significantly different bacterial taxa in the study participants. Statistical analyses were performed using SPSS version 20 (IBM's Corporate Privacy Office, New York, NY, USA).

## Results

Overall, we tested 46 volunteers, including 9 normal weight and 9 obese individuals from Saudi Arabia and 16 normal weight and 12 obese individuals from France. The BMI of the obese Saudis and obese French was significantly higher than that of normal weight French (*P=*0.001 and *P*=0.02, respectively) and Saudis (*P=*0.003 and *P*=0.03, respectively). No difference existed regarding the age among the groups of participants (Table 1).

### Composition of Gut microbiota

**Figure 1:**
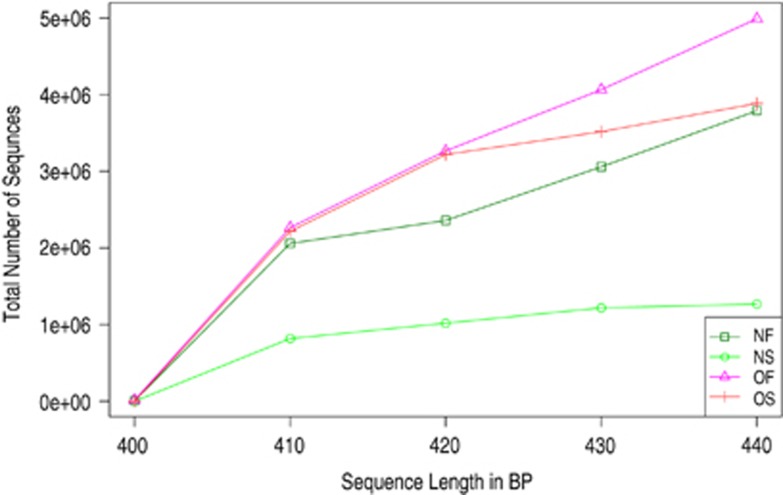
Length and total sequencing reads for the group tested. OF, obese French; OS, obese Saudis; NF, normal French; NS, normal Saudis.

**Figure 2:**
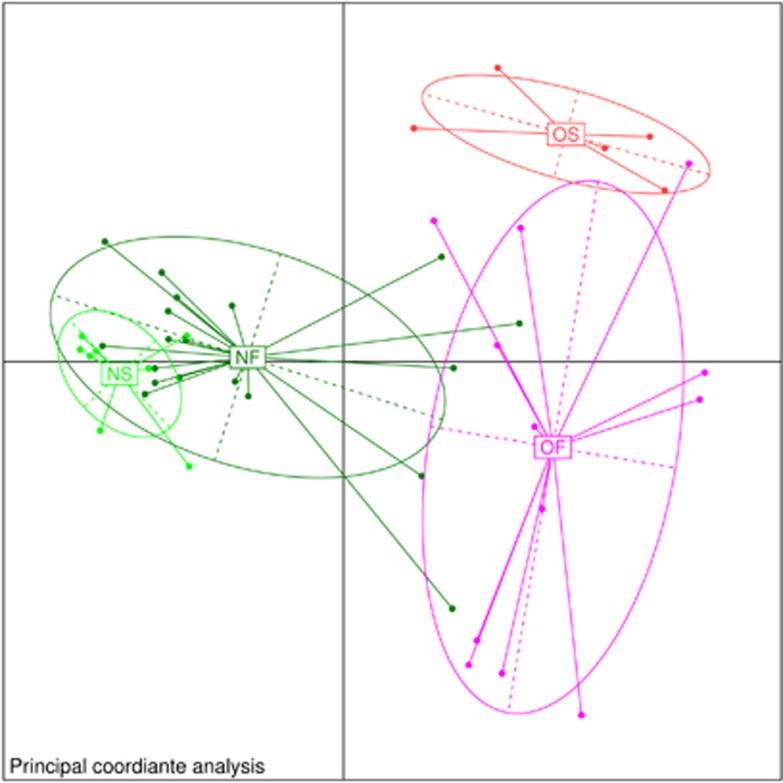
Principle coordinate analysis of the overall composition of the genera communities among the four groups. OF, obese French; OS, obese Saudis; NF, normal French; NS, normal Saudis.

We obtained approximately 14 million 16S rRNA gene sequence reads of the V3–V4 region, corresponding to 27% reads for normal weight French, 36% for obese French, 28% for obese Saudis and 9% for normal weight Saudis (Figure 1). The analysis of the high-quality trimmed reads revealed that the gut microbiota of obese and normal weight subjects contained sequences from 14 different bacterial divisions/phyla (Supplementary Figure S1). Most of the sequences belonged to *Firmicutes* and *Actinobacteria* followed by *Proteobacteria*, *Bacteroidetes* and *Verrucomicrobia* and were present in all participants. Moreover, 315 different genera from all sequence reads were identified and a Principle Coordinate analysis was performed to compare the overall composition of the genera communities between the groups (Figure 2). Principle Coordinate analysis showed that obese and normal weight individuals clustered independently. Normal weight individuals clustered together, but obese Saudis clustered independently from obese French.

### Common bacterial core

**Figure 3:**
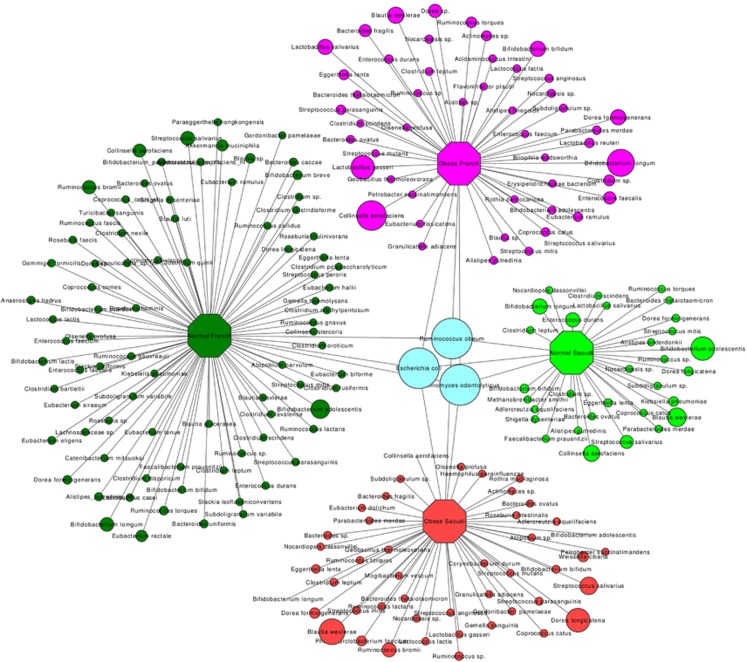
Network of bacterial species core among the individuals tested.

We detected 1357 different species in all the individuals tested. Obese French individuals had 689 different species, normal weight French had 888 species, obese Saudis 355 species and normal weight Saudis 346 species. Among these, 367 species were only detected in normal weight French and 186 species were only detected in obese French, whereas 43 species were only present in obese Saudis and 34 only in normal weight Saudis (Supplementary Figure S2). Statistical analysis showed that normal weight French had more species than obese French (*P*=0.02), normal weight Saudis (*P*=0.003) and obese Saudis (*P*=0.004). Obese French had more species than normal weight Saudis (*P*=0.005) and obese Saudis (*P*=0.01), and no difference existed between normal weight and obese Saudis. At least 50% of obese and normal weight Saudis had 23 common species, whereas 50% of obese and normal weight French possessed 29 common species (Supplementary Figures S3 and S4). However, when we compared all the individuals for both countries, we detected only three species (*Actinomyces odontolyticus*, *Escherichia coli* and *Ruminococcus obeum*) in at least 50% of them. Moreover, we detected 34 species in >50% of normal weight Saudis, 49 species in >50% of obese Saudis, 52 in >50% of obese French and 88 species in >50% of normal weight French (Figure 3 and Supplementary Table S1).

### Gut microbiota alterations among the different groups

#### French versus Saudis

For the different bacterial divisions/phyla, statistical analysis indicated that French individuals had significantly more *Verrucomicrobia* than Saudis (*P*=0.01). Moreover, *Fusobacteria* were only detected in French individuals, whereas no difference existed for the other phyla (Supplementary Figure S5). Genera analysis showed that French individuals had significantly more *Bifidobacterium* than Saudis (*P*=0.01; Supplementary Figure S6). No difference was found for *Prevotella*, *Streptococcus* and *Actinomyces*, and no difference was found for *Bacteroides thetaiotaomicron*, *Methanobrevibacter smithii*, *Bifidobacterium longum*, *Clostridium leptum*, *Prevotella copri* and other species associated with weight modifications between normal weight French and obese Saudis (Supplementary Figure S7). Interestingly, *Lactobacillus reuteri* was detected only in obese French and *Lactobacillus sakei* only in French individuals, whereas obese Saudis did not have it at all.

#### Obese versus normal weight French

Obese French presented significantly more relative *Proteobacteria* (*P*=0.002) and *Bacteroidetes* (*P*=0.05) than normal weight French (Supplementary Figure S5). Sequences belonging to *Spirochaetae* and *Elusimicrobia* were only detected in normal weight French, whereas sequences belonging to *Gemmatimonadetes* were only detected in obese French. Genera analysis indicated that obese French possessed significantly more relative *Lactobacillus* (*P*=0.05), *Escherichia*–*Shigella* (*P*=0.01) and *Bacteroides* (*P*=0.05) and significantly less *Clostridium* (*P*=0.02) and *Faecalibacterium* (*P*=0.001) than normal weight French (Supplementary Figure S6). Statistical analysis for species revealed obese French had more *Bacteroides fragilis* (*P*=0.05), *Blautia wexlerae* (*P*=0.05) and *E. coli* than normal weight French (*P*=0.02). In contrast, obese French possessed significantly less relative *Bifidobacterium adolescentis* (*P*=0.0002), *Bifidobacterium breve* (*P*=0.0003) and *Ruminococcus lactaris* (*P*=0.001). *Lactobacillus gasseri* and *L. reuteri* were detected only in obese French.

#### Obese French versus obese Saudis

Obese French had significantly more relative *Verrucomicrobia* than obese Saudis (*P*=0.001; Supplementary Figure S5). For genera, obese French possessed significantly less *Faecalibacterium* (*P*=0.04), *Blautia* (*P*=0.05) and *Bifidobacterium* (*P*=0.03) than obese Saudis. Among the compared species (Supplementary Figure S7), obese French had more relative *Rothia mucilaginosa* and *Ruminococcus bromii* than obese Saudis (*P*=0.006 and *P*=0.03, respectively).

#### Obese French versus normal weight Saudis

Obese French possessed significantly less relative *Ruminococcus* (*P*=0.03) and more *Verrucomicrobia* (*P*=0.02) than normal weight Saudis (Supplementary Figure S5). For genera, obese French possessed significantly less *Faecalibacterium* than normal weight Saudis (*P*=0.03), whereas species statistical analysis indicated that obese French had more *B. fragilis* (*P*=0.05) and less *B. adolescentis* (*P*=0.01) than normal weight Saudis. Finally, *Bifidobacterium bifidum* was represented significantly more in obese French than in normal weight Saudis (*P*=0.05).

#### Normal weight French versus obese Saudis

No difference was found in phyla between normal weight French and obese Saudis (Supplementary Figure S5). Regarding genera, normal weight French had significantly more relative *Clostridium* (*P*=0.04) and less *Dorea* (*P*=0.004) than obese Saudis (Supplementary Figure S6). Normal weight French possessed significantly more *B. breve* (*P*=0.0002) and significantly less *B. wexlerae* (*P*=0.02) and *R. mucilaginosa* (*P*=0.006) than obese Saudis.

#### Obese versus normal weight Saudis

For the different bacterial phyla, obese Saudis possessed significantly more relative *Firmicutes* than normal weight Saudis (*P*=0.001; Supplementary Figure S5). For genera, obese Saudis had significantly more relative *Dorea* than normal weight Saudis (*P*=0.004), whereas no significant difference was found for species.

#### Normal weight French versus Saudis

Normal weight Saudis possessed significantly more relative *Proteobacteria* than normal weight French (*P*=0.02; Supplementary Figure S5). Regarding genera, normal weight French had significantly more relative *B. breve* and significantly less *B. wexlerae* than normal weight Saudis (*P*=0.0004 and *P*=0.02, respectively; Supplementary Figure S6).

### Microbial richness and biodiversity

**Figure 4:**
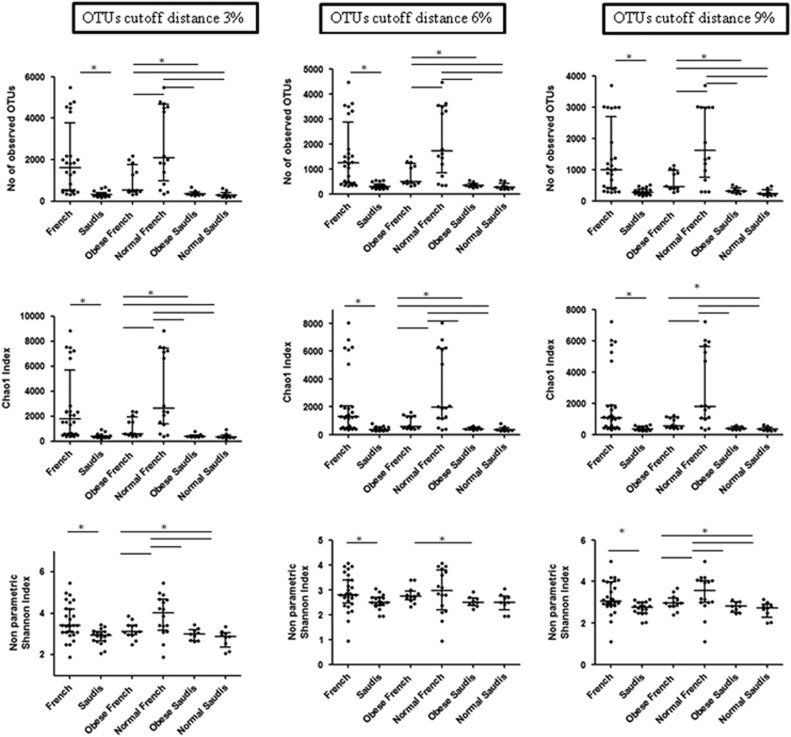
Boxplots of the observed OTUs, the Chao1 indexes and the non-parametric Shannon indexes at OTU cutoffs of 3, 6 and 9 distance units.

We then compared microbial richness, estimated by the Chao1 index, and biodiversity, assessed by a non-parametric Shannon index for comparison among the groups. In our calculations, we took into account different OTU distance unit cutoffs, namely 3, 6 and 9. Using the non-parametric Kruskal–Wallis test for comparisons, we found that French individuals had significantly higher richness and biodiversity than obese Saudis at all the OTU cutoffs (*P*<0.05; Figure 4). Moreover, obese French had significantly lower richness and biodiversity than normal weight weight French at all the OTU cutoffs (*P*<0.05). Obese French also had significantly higher richness and biodiversity than obese and normal weight Saudis. Similarly, normal weight French had significantly higher richness and biodiversity than obese and normal weight Saudis. Microbial richness and biodiversity was not different between obese and normal Saudis at all the OTU cutoffs (Figure 4).

## Discussion

In this study, we used Illumina Miseq deep sequencing to explore the gut microbiota of obese and normal weight people from France and Saudi Arabia. We found that obese French had lower richness and biodiversity than normal weight French, whereas this difference was not found between obese and normal weight Saudis. Moreover, we found that obese French possessed more *Bacteroidetes* than normal weight French, whereas obese Saudis possessed more *Firmicutes* but had no difference in *Bacteroidetes* compared with normal weight Saudis. As our study was the first attempt to evaluate the statistical variability of the gut microbiota among Saudis, it should be considered a pilot study. In any case, our sample size was comparable to that of previous influential studies.^23, 24^ A limitation of our study was that we tested only male volunteers from Saudi Arabia and that we did not measure the metabolic activity of the microbiota by metagenomic analysis. Saudi Arabia has a much conserved society and local people are reluctant to volunteer for studies involving stool samples, in particular females. There is need of further studies to investigate the relationship between obesity and gut microbiota among Saudi female population. The nature of changes in the gut microbiota associated with obesity is a subject of controversy. Indeed, our data on the gut microbiota from participants of countries with different cultural and dietary traditions revealed discrepancies in the composition of the gut microbiota. Previously, obesity has been associated either with a reduction^15, 24, 25, 26, 27^ or with an increase^28, 29, 30, 31^ of *Bacteroidetes*. Contrasting data on the composition of the gut microbiota can be found even in different analyses originating from the same laboratory.^8, 32^ These discrepancies in data suggest that gut microbiota studies may suffer from biases due to subject selection, the evolution of molecular techniques, DNA extraction and amplification methods and sequencing technologies.^33, 34, 35^ This is also highlighted by the discrepancies of molecular analysis and analyses by microbial culture of patient stool samples.^36^

Obese French presented significantly more *Lactobacillus* than normal weight French. Moreover, *L. reuteri* was only present in obese French and *L. sakei* only in French individuals. It is possible that the increased consumption of fermented dairy products and probiotics containing *Lactobacillus* sp. can explain this difference.^37, 38^ There is evidence that *Lactobacillus* sp. are associated with weight modifications,^1, 39^ and in a recent comparative genomics analysis of *Lactobacillus* sp., we found that weight gain associated with *Lactobacillus* spp. resulted in a limited ability to break down fructose or glucose and might reduce ileal brake effects.^40^ In addition, *L. reuteri* has been previously associated with weight modifications in humans^7^ and in mice.^41^ Moreover, the presence of *L. gasseri* was positively correlated with the BMI in diabetic and non-diabetic women.^42^ In addition, the *L. reuteri* population was increased in children with Kwashiorkor under treatment with ready-to-use therapeutic food.^27^

Saudis had significantly lower diversity in their diets compared with that of French individuals. Rapid economic growth in the past few decades has drastically changed the lifestyle and food habits in Saudi Arabia.^43, 44^ Particularly in the urban areas, population shift away from traditional food to Western cuisines. Many studies commonly reported irregular meal habit, regular consumption of snacks mainly made from junk foods, eating away from home and abundance use of carbonated beverages in Saudi population.^43, 44^ In a recent study from Jeddah was found a 87% daily consumption of snacks and that the 85% of youth Saudis depended on fast foods like shawarma, hamburger, pizza and fried chicken. In contrast, a French diet is rich in protein and in fermented dairy products.^37, 38^ The differences between the French and Saudi microbiomes can be related to the differences in diet, and the poor variety of food in Saudi Arabia can most likely explain the poor biodiversity of the Saudi microbiome.

Diet has a critical role in the gut microbiota, as demonstrated by the fact that bacterial species associated with a high-fat, high-sugar diet promote obesity in gnotobiotic mice.^45^ In mammals, both diet and phylogeny influence the increase in bacterial diversity from carnivore to omnivore to herbivore.^46^ Moreover, the gut microbiota can rapidly respond to altered diet, potentially facilitating the diversity of human dietary lifestyles.^4, 6^ A high-fiber diet has been associated with an enrichment of the microbiome of children from rural Africa compared with children from Europe,^9^ and differences associated with diet were found in the gut microbiota of Americans compared with Malawi and Amerindian populations.^47^ Moreover, the gut microbiota of Colombians was significantly different from that of Americans, Europeans and Asians,^23^ whereas another recent study, in which the gut microbiota of Hazdas, Burkinabes, Malawians, Italians and Americans was compared, found that geography was clearly the most important grouping factor.^48^ In agreement with these studies, we here show that the origin of the population explains more variability in the composition of the gut microbiota than factors such as BMI or gender and is very difficult to make conclusions about the association of the gut microbiota and obesity.

In conclusion, this study explores the gut microbiota of Saudis and reveals that the cultural and dietary traditions of these people result in an important decrease of the richness of their microbiota. The discrepancy of the gut microbiota comparison between obese and normal weight individuals from the two countries indicates that it is very difficult to make conclusions about the association of the gut microbiota and obesity. Indeed, there are large discrepancies in the literature among the results of studies testing the role of gut microbiota in obesity.^15, 24, 25, 26, 27, 28, 29, 30, 31^ To understand how cultural and dietary habits are changing the microbiota, we emphasize the importance of sampling populations of humans from different geographical regions and cultural traditions to explore features of the gut microbiota that are unique to different locations and lifestyles.

## Figures and Tables

**Table 1:** Patients characteristics

*Subjects*	*Normal Saudis*	*Obese Saudis*	*Normal French*	*Obese French*
Age (median±s.d.)	28±4	26±3	34±5	39±13
Sex (percentage of males)	100%	100%	44%	58%
Body mass index (median±s.d.)	24.5±3.2	46.0±5.9		38.3±7.9
