# Supplementary material for: Comparison of the gut microbiota of people in France and Saudi Arabia
Source: Nutr Diabetes. 2015 Apr 27;5(4):e153–. doi: 10.1038/nutd.2015.3 (PMC4423199; doi:10.1038/nutd.2015.3)
Supplement: Supplementary Information [file nutd20153x1.doc]

**Supplementary Figure 1**.

**
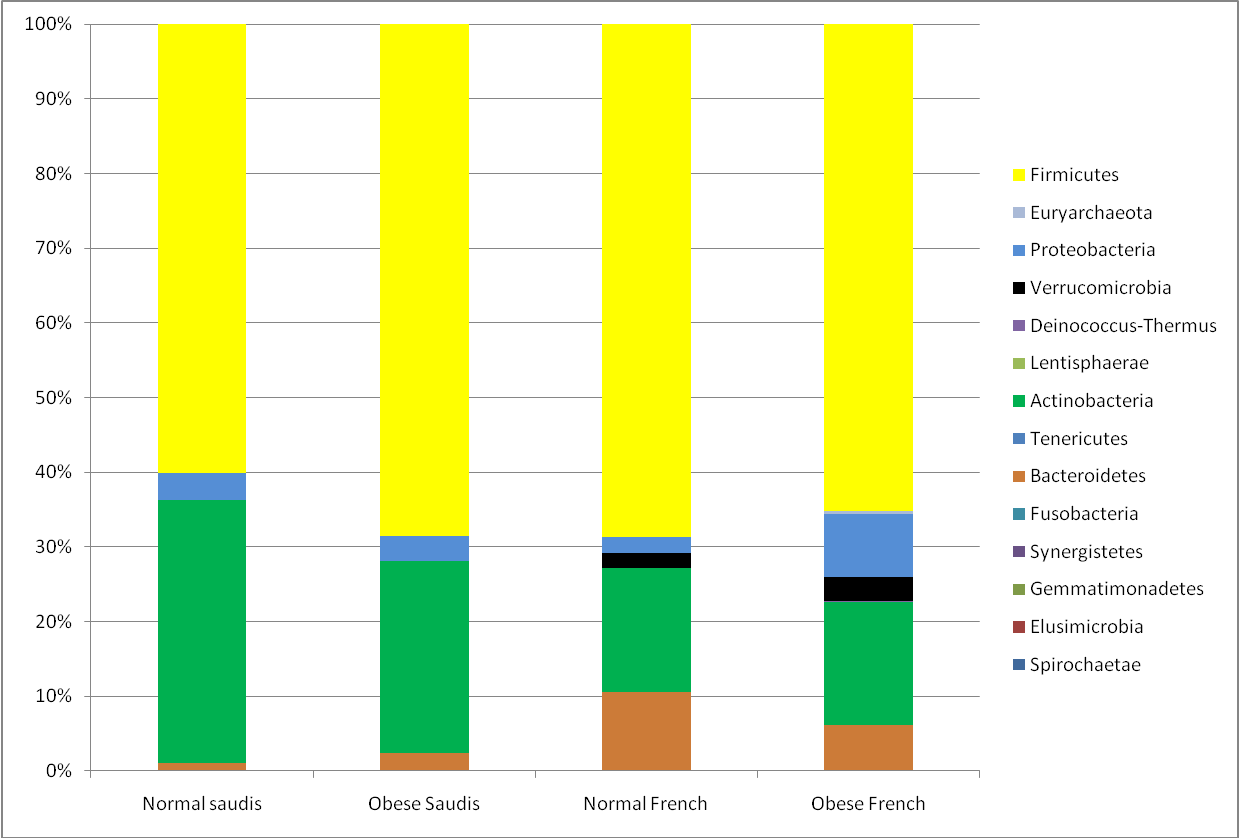
**

**Supplementary Figure 2**.

**Supplementary Figure 3**.

**
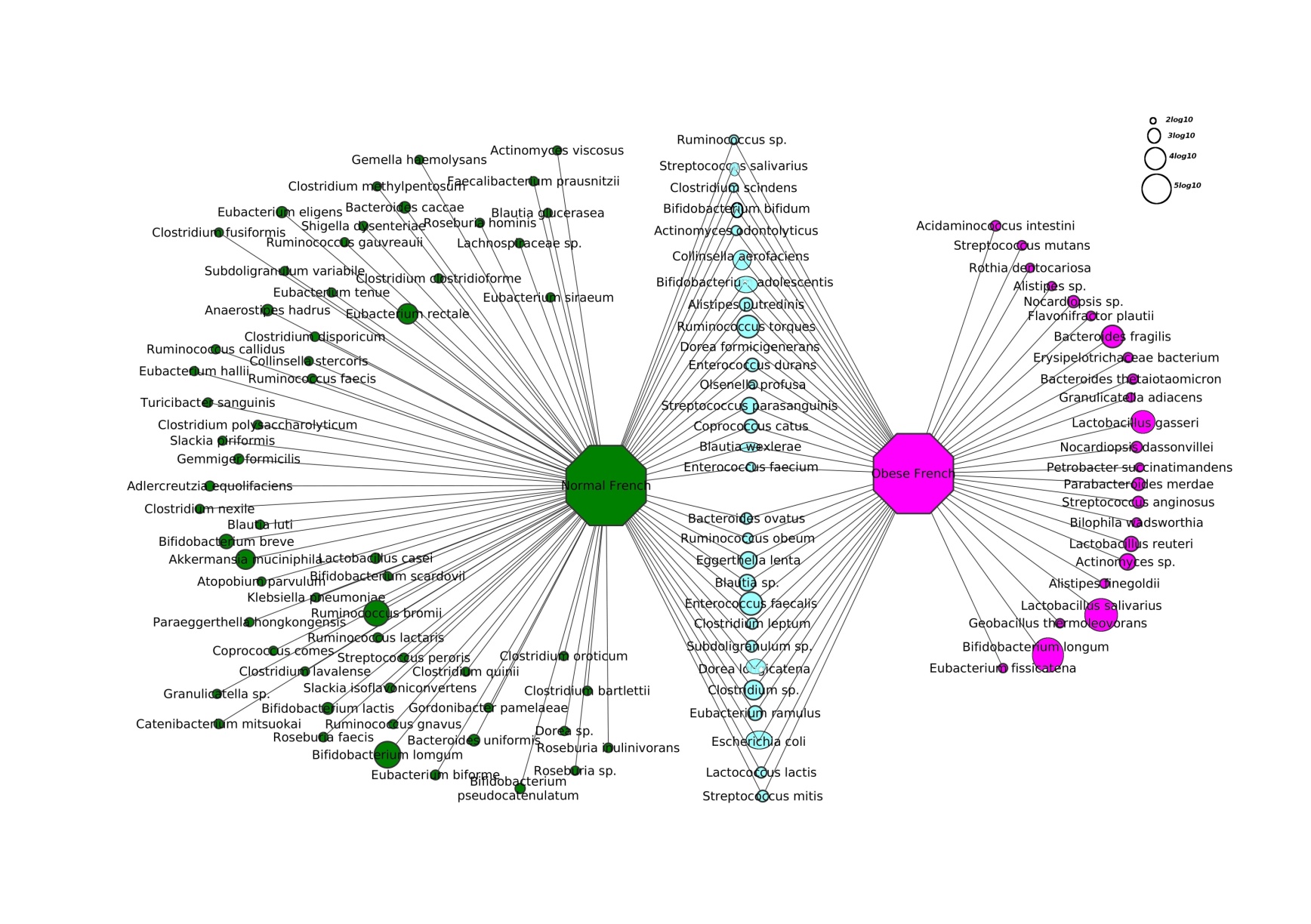
**

**Supplementary Figure 4**.

**
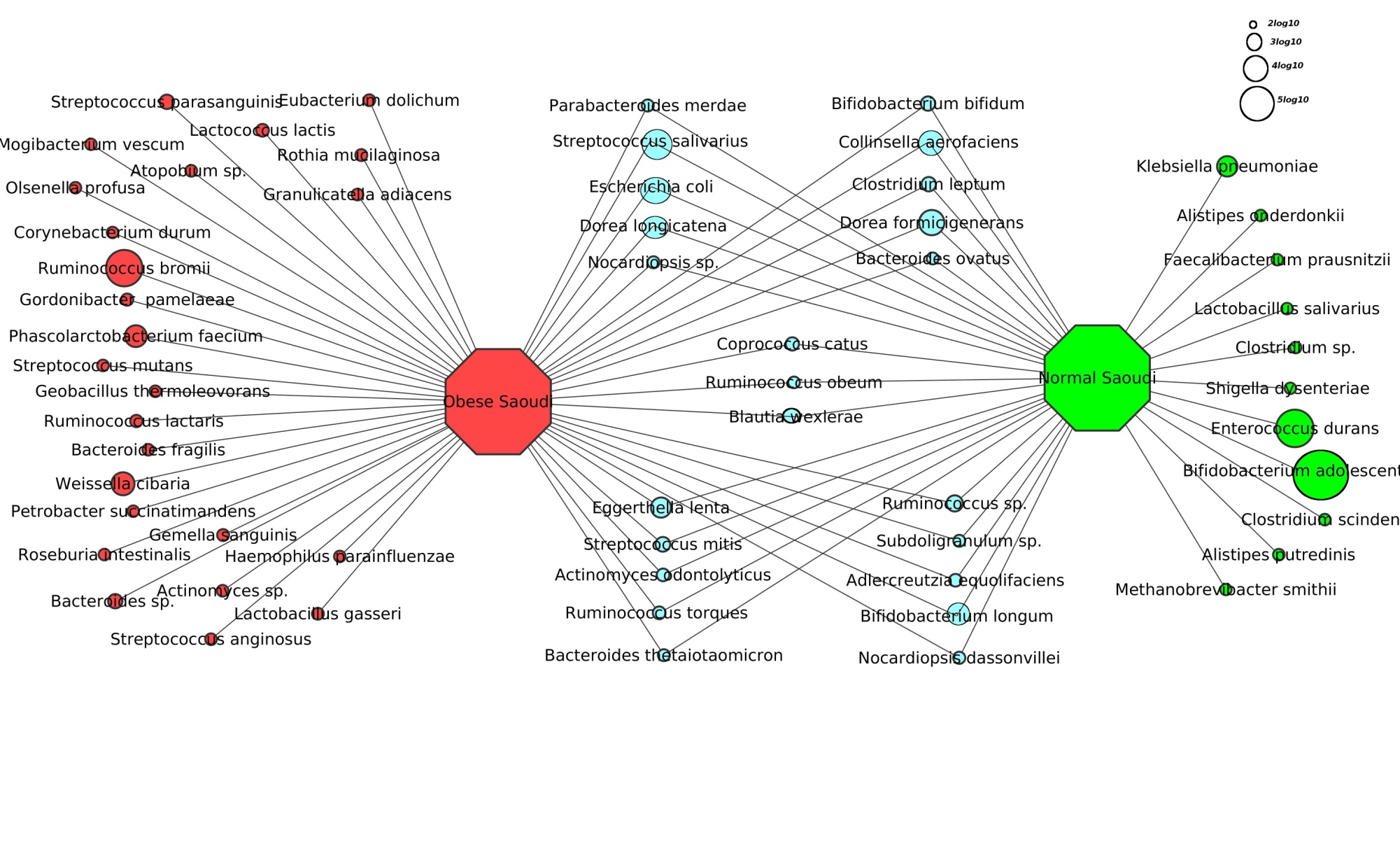
**

**Supplementary Figure 5**.

**Supplementary Figure 6**.

**Supplementary Figure 7**.


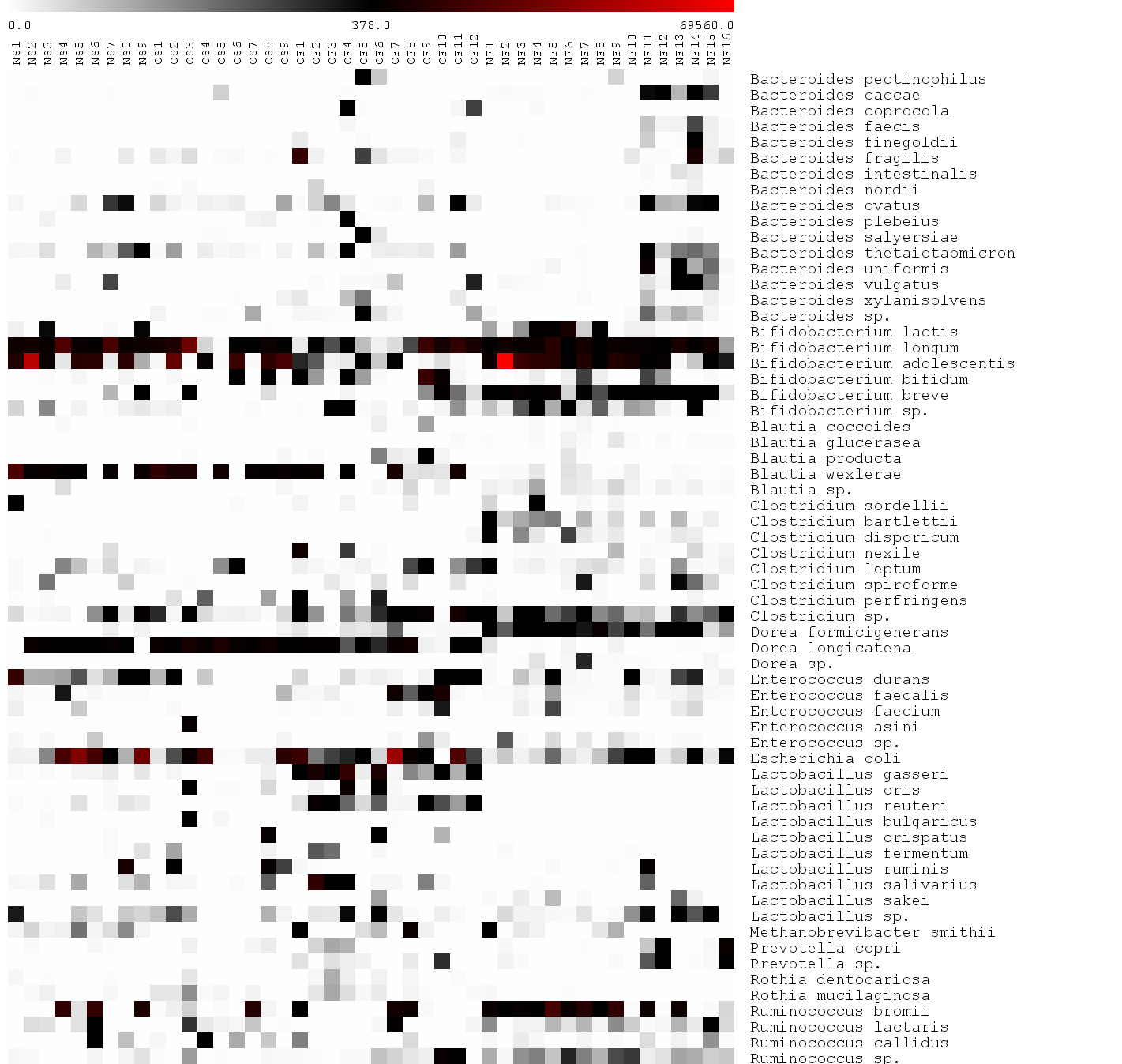


**Supplementary Table 1. Core species for the four groups tested**

| **Normal weight Saudis** | **No of**  **sequences** | **Obese French** | **No of**  **sequences** | **Obese Saudis** | **No of**  **sequences** | **Normal weight French** | **No of**  **sequences** |
| --- | --- | --- | --- | --- | --- | --- | --- |
| *Actinomyces odontolyticus* | 1614 | *Acidaminococcus intestini* | 5332 | *Actinomyces odontolyticus* | 1552 | *Actinomyces odontolyticus* | 1438 |
| *Adlercreutzia equolifaciens* | 38 | *Actinomyces odontolyticus* | 1897 | *Actinomyces* sp. | 592 | *Actinomyces viscosus* | 114 |
| *Alistipes onderdonkii* | 300 | *Actinomyces sp.* | 25239 | *Adlercreutzia equolifaciens* | 3057 | *Adlercreutzia equolifaciens* | 3255 |
| *Alistipes putredinis* | 359 | *Alistipes finegoldii* | 1722 | *Atopobium sp.* | 674 | *Akkermansia muciniphila* | 36189 |
| *Bacteroides ovatus* | 297 | *Alistipes putredinis* | 6408 | *Bacteroides fragilis* | 469 | *Alistipes putredinis* | 9475 |
| *Bacteroides thetaiotaomicron* | 287 | *Alistipes sp.* | 609 | *Bacteroides ovatus* | 953 | *Anaerostipes hadrus* | 7914 |
| *Bifidobacterium adolescentis* | 235931 | *Bacteroides fragilis* | 45853 | *Bacteroides sp.* | 7214 | *Atopobium parvulum* | 90 |
| *Bifidobacterium bifidum* | 33 | *Bacteroides ovatus* | 7241 | *Bacteroides thetaiotaomicron* | 961 | *Bacteroides caccae* | 8466 |
| *Bifidobacterium longum* | 72660 | *Bacteroides thetaiotaomicron* | 4746 | *Bifidobacterium adolescentis* | 329965 | *Bacteroides ovatus* | 1267 |
| *Blautia wexlerae* | 109628 | *Bifidobacterium adolescentis* | 7498 | *Bifidobacterium bifidum* | 7160 | *Bacteroides uniformis* | 8572 |
| *Clostridium leptum* | 157 | *Bifidobacterium bifidum* | 74514 | *Bifidobacterium longum* | 246778 | *Bifidobacterium adolescentis* | 104450 |
| *Clostridium scindens* | 503 | *Bifidobacterium longum* | 182624 | *Blautia wexlerae* | 169831 | *Bifidobacterium bifidum* | 1224 |
| *Clostridium sp.* | 121 | *Bilophila wadsworthia* | 1300 | *Clostridium leptum* | 7224 | *Bifidobacterium breve* | 18230 |
| *Collinsella aerofaciens* | 99614 | *Blautia sp.* | 2651 | *Collinsella aerofaciens* | 241577 | *Bifidobacterium lactis* | 9379 |
| *Coprococcus catus* | 234 | *Blautia wexlerae* | 82599 | *Coprococcus catus* | 4299 | *Bifidobacterium lomgum* | 59403 |
| *Dorea formicigenerans* | 5409 | *Clostridium leptum* | 6906 | *Corynebacterium durum* | 74 | *Bifidobacterium scardovii* | 235 |
| *Dorea longicatena* | 17212 | *Clostridium scindens* | 742 | *Dorea formicigenerans* | 29013 | *Bifidobacterium pseudocatenulatum* | 4082 |
| *Eggerthella lenta* | 9063 | *Clostridium sp.* | 34814 | *Dorea longicatena* | 151177 | *Blautia glucerasea* | 145 |
| *Enterococcus durans* | 64905 | *Collinsella aerofaciens* | 204963 | *Eggerthella lenta* | 12641 | *Blautia luti* | 410 |
| *Escherichia coli* | 21527 | *Coprococcus catus* | 14964 | *Escherichia coli* | 125554 | *Blautia* sp. | 23045 |
| *Faecalibacterium prausnitzii* | 32 | *Dorea formicigenerans* | 1807 | *Eubacterium dolichum* | 819 | *Blautia wexlerae* | 184 |
| *Klebsiella pneumoniae* | 22391 | *Dorea formicigenerans* | 55532 | *Gemella sanguinis* | 1362 | *Catenibacterium mitsuokai* | 1686 |
| *Lactobacillus salivarius* | 263 | *Dorea longicatena* | 74317 | *Geobacillus thermoleovorans* | 439 | *Clostridium bartlettii* | 1404 |
| *Methanobrevibacter smithii* | 88 | *Eggerthella lenta* | 27160 | *Gordonibacter pamelaeae* | 1817 | *Clostridium clostridioforme* | 390 |
| *Nocardiopsis dassonvillei* | 336 | *Enterococcus durans* | 13066 | *Granulicatella adiacens* | 648 | *Clostridium disporicum* | 1305 |
| *Nocardiopsis sp.* | 292 | *Enterococcus faecalis* | 47845 | *Haemophilus parainfluenzae* | 189 | *Clostridium fusiformis* | 76 |
| *Parabacteroides merdae* | 972 | *Enterococcus faecium* | 1027 | *Lactobacillus gasseri* | 1047 | *Clostridium lavalense* | 120 |
| *Ruminococcus obeum* | 341 | *Erysipelotrichaceae bacterium* | 3787 | *Lactococcus lactis* | 3287 | *Clostridium leptum* | 829 |
| *Ruminococcus sp.* | 681 | *Escherichia coli* | 363367 | *Mogibacterium vescum* | 155 | *Clostridium methylpentosum* | 114 |
| *Ruminococcus torques* | 2925 | *Eubacterium fissicatena* | 978 | *Nocardiopsis dassonvillei* | 1332 | *Clostridium nexile* | 381 |
| *Shigella dysenteriae* | 38 | *Eubacterium ramulus* | 18663 | *Nocardiopsis sp.* | 1201 | *Clostridium oroticum* | 252 |
| *Streptococcus mitis* | 1536 | *Flavonifractor plautii* | 2019 | *Olsenella profusa* | 270 | *Clostridium polysaccharolyticum* | 93 |
| *Streptococcus salivarius* | 53618 | *Geobacillus thermoleovorans* | 1094 | *Parabacteroides merdae* | 620 | *Clostridium quinii* | 81 |
| *Subdoligranulum* sp. | 684 | *Granulicatella adiacens* | 1098 | *Petrobacter succinatimandens* | 186 | *Clostridium scindens* | 369 |
|  |  | *Lactobacillus gasseri* | 148809 | *Phascolarctobacterium faecium* | 25788 | *Clostridium* sp. | 1513 |
|  |  | *Lactobacillus reuteri* | 20794 | *Roseburia intestinalis* | 285 | *Collinsella aerofaciens* | 53061 |
|  |  | *Lactobacillus salivarius* | 73745 | *Rothia mucilaginosa* | 1271 | *Collinsella stercoris* | 167 |
|  |  | *Lactococcus lactis* | 5995 | *Ruminococcus bromii* | 62227 | *Coprococcus catus* | 1265 |
|  |  | *Nocardiopsis dassonvillei* | 7961 | *Ruminococcus lactaris* | 2296 | *Coprococcus comes* | 336 |
|  |  | *Nocardiopsis sp.* | 10691 | *Ruminococcus obeum* | 198 | *Dorea formicigenerans* | 13210 |
|  |  | *Olsenella profusa* | 130 | *Ruminococcus sp.* | 11211 | *Dorea longicatena* | 106 |
|  |  | *Parabacteroides merdae* | 13264 | *Ruminococcus torques* | 507 | *Dorea sp.* | 355 |
|  |  | *Petrobacter succinatimandens* | 327 | *Ruminococcus obeum* | 189 | *Eggerthella lenta* | 618 |
|  |  | *Rothia dentocariosa* | 902 | *Streptococcus anginosus* | 640 | *Enterococcus durans* | 2607 |
|  |  | *Ruminococcus obeum* | 2659 | *Streptococcus mitis* | 6630 | *Enterococcus faecalis* | 296 |
|  |  | *Ruminococcus sp.* | 1861 | *Streptococcus mutans* | 498 | *Enterococcus faecium* | 375 |
|  |  | *Ruminococcus torques* | 31949 | *Streptococcus parasanguinis* | 7494 | *Escherichia coli* | 6568 |
|  |  | *Streptococcus anginosus* | 10151 | *Streptococcus salivarius* | 94345 | *Eubacterium biforme* | 2809 |
|  |  | *Streptococcus mitis* | 9078 | *Subdoligranulum* sp*.* | 628 | *Eubacterium eligens* | 7062 |
|  |  | *Streptococcus mutans* | 2188 | *Weissella cibaria* | 28781 | *Eubacterium hallii* | 346 |
|  |  | *Streptococcus parasanguinis* | 22529 |  |  | *Eubacterium ramulus* | 1117 |
|  |  | *Streptococcus salivarius* | 332090 |  |  | *Eubacterium rectale* | 38210 |
|  |  | *Subdoligranulum* sp. | 8094 |  |  | *Eubacterium siraeum* | 699 |
|  |  |  |  |  |  | *Eubacterium tenue* | 289 |
|  |  |  |  |  |  | *Faecalibacterium prausnitzii* | 782 |
|  |  |  |  |  |  | *Gemella haemolysans* | 89 |
|  |  |  |  |  |  | *Gemmiger formicilis* | 3860 |
|  |  |  |  |  |  | *Gordonibacter pamelaeae* | 717 |
|  |  |  |  |  |  | *Granulicatella sp.* | 250 |
|  |  |  |  |  |  | *Klebsiella pneumoniae* | 104 |
|  |  |  |  |  |  | *Lachnospiraceae sp.* | 959 |
|  |  |  |  |  |  | *Lactobacillus casei* | 4543 |
|  |  |  |  |  |  | *Lactococcus lactis* | 1428 |
|  |  |  |  |  |  | *Olsenella profusa* | 309 |
|  |  |  |  |  |  | *Paraeggerthella hongkongensis* | 274 |
|  |  |  |  |  |  | *Roseburia faecis* | 1580 |
|  |  |  |  |  |  | *Roseburia hominis* | 97 |
|  |  |  |  |  |  | *Roseburia inulinivorans* | 1655 |
|  |  |  |  |  |  | *Roseburia sp.* | 497 |
|  |  |  |  |  |  | *Ruminococcus bromii* | 56421 |
|  |  |  |  |  |  | *Ruminococcus callidus* | 482 |
|  |  |  |  |  |  | *Ruminococcus faecis* | 1296 |
|  |  |  |  |  |  | *Ruminococcus gauvreauii* | 106 |
|  |  |  |  |  |  | *Ruminococcus gnavus* | 81 |
|  |  |  |  |  |  | *Ruminococcus lactaris* | 1959 |
|  |  |  |  |  |  | *Ruminococcus obeum* | 2942 |
|  |  |  |  |  |  | *Ruminococcus sp.* | 704 |
|  |  |  |  |  |  | *Ruminococcus torques* | 14218 |
|  |  |  |  |  |  | *Shigella dysenteriae* | 533 |
|  |  |  |  |  |  | *Slackia isoflavoniconvertens* | 2323 |
|  |  |  |  |  |  | *Slackia piriformis* | 252 |
|  |  |  |  |  |  | *Streptococcus mitis* | 197 |
|  |  |  |  |  |  | *Streptococcus parasanguinis* | 3161 |
|  |  |  |  |  |  | *Streptococcus peroris* | 121 |
|  |  |  |  |  |  | *Streptococcus salivarius* | 60415 |
|  |  |  |  |  |  | *Subdoligranulum sp.* | 1495 |
|  |  |  |  |  |  | *Subdoligranulum variabile* | 262 |
|  |  |  |  |  |  | *Turicibacter sanguinis* | 916 |
