# Supplementary material for: Comparison of the gut microbiota of people in France and Saudi Arabia
Source: Nutr Diabetes. 2015 Apr 27;5(4):e153–. doi: 10.1038/nutd.2015.3 (PMC4423199; doi:10.1038/nutd.2015.3)
Supplement: Supplementary Figure Legends [file nutd20153x2.doc]

**Supplementary Figures.**

**Supplementary Figure 1**. The relative abundance of the gut microbiota phyla among the groups tested .

**Supplementary Figure 2**. Network of species diversity obtained for the four groups tested

**Supplementary Figure 3**. Network of bacterial species core among French individuals

**Supplementary Figure 4**. Network of bacterial species core among Saudis individuals

**Supplementary Figure 5**. Scatter plots at the phylum levels.

**p*>0.05; The medians and the interquartile ranges are shown.

**Supplementary Figure 6**. Scatter plots at the genus levels.

**p*>0.05, The medians and the interquartile ranges are shown.

**Supplementary Figure 7**. Bacterial species associated with weight modifications for the individuals tested

**Supplementary information is available at International Journal of Obesity's website**
